# Identification of Trypanosome Proteins in Plasma from African Sleeping Sickness Patients Infected with *T. b. rhodesiense*

**DOI:** 10.1371/journal.pone.0071463

**Published:** 2013-08-08

**Authors:** Brett A. Eyford, Rushdy Ahmad, John C. Enyaru, Steven A. Carr, Terry W. Pearson

**Affiliations:** 1 Department of Biochemistry and Microbiology, University of Victoria, Victoria, British Columbia, Canada; 2 The Broad Institute of MIT and Harvard, Cambridge, Massachusetts, United States of America; 3 Department of Biochemistry, Makerere University, Kampala, Uganda; Louisiana State University, United States of America

## Abstract

Control of human African sleeping sickness, caused by subspecies of the protozoan parasite *Trypanosoma brucei*, is based on preventing transmission by elimination of the tsetse vector and by active diagnostic screening and treatment of infected patients. To identify trypanosome proteins that have potential as biomarkers for detection and monitoring of African sleeping sickness, we have used a ‘deep-mining” proteomics approach to identify trypanosome proteins in human plasma. Abundant human plasma proteins were removed by immunodepletion. Depleted plasma samples were then digested to peptides with trypsin, fractionated by basic reversed phase and each fraction analyzed by liquid chromatography-tandem mass spectrometry (LC-MS/MS). This sample processing and analysis method enabled identification of low levels of trypanosome proteins in pooled plasma from late stage sleeping sickness patients infected with *Trypanosoma brucei rhodesiense.* A total of 254 trypanosome proteins were confidently identified. Many of the parasite proteins identified were of unknown function, although metabolic enzymes, chaperones, proteases and ubiquitin-related/acting proteins were found. This approach to the identification of conserved, soluble trypanosome proteins in human plasma offers a possible route to improved disease diagnosis and monitoring, since these molecules are potential biomarkers for the development of a new generation of antigen-detection assays. The combined immuno-depletion/mass spectrometric approach can be applied to a variety of infectious diseases for unbiased biomarker identification.

## Introduction

Several methods are currently used to diagnose human African trypanosomiasis (HAT), also known as African sleeping sickness. These include detection of anti-trypanosome antibodies [1], [2], amplification of DNA sequences [3], [4] and direct observation of parasites by microscopic examination of patient blood or cerebrospinal fluid (CSF), usually preceded by parasite enrichment techniques [5]. Although each of these methods has problems that hinder reliable, high throughput and cost effective disease diagnosis, together, they do help disease control efforts [6].

Currently, the only way to definitively diagnose HAT in the field is to microscopically observe trypanosomes in the blood (early stage disease) and in the CSF (late stage disease). Using parasite enrichment techniques, the current limit of microscopic detection is ∼100 trypanosomes/mL of blood [5], thus between parasitemic waves, parasite numbers lower than this make microscopic detection unreliable. Due to low sensitivity and low throughput, microscopic diagnosis is only used to confirm suspected infections and is not an effective tool for mass screening campaigns.

The card agglutination test for trypanosomiasis (CATT, [1]) is the most commonly used assay for mass screening in the field as it is relatively easily performed and requires minimal instrumentation [5]. It is not ideal, since it is only useful for detecting antibodies generated during infections with *Trypanosoma brucei gambiense* that often, but not always, express a particular, defined variant surface glycoprotein (VSG) during waves of parasitemias. Infections with *T. b. gambiense* parasites in west and central Africa may be missed if that VSG type is not expressed. In addition, the CATT is not useful for detecting infections with *T. b. rhodesiense*, parasites that cause acute infections in eastern and southern Africa.

Although antibody detection is often used as a first line diagnostic test for many infectious diseases, antibodies often persist in the bloodstream after a person has been cured. Therefore antibody detection is usually not useful for discriminating between current and past infections. For this reason, and the fact that no antibody detection tests for *T. b. rhodesiense* infections are available, diagnostic assays for African sleeping sickness based on parasite antigen detection are deemed to be more desirable. Previous studies have shown that trypanosome antigens are detectable by immunoassay in the sera of infected cattle [7], [8], [9], rodents [10], vervet monkeys [11] and humans [12], [13]. Previous work from our lab showed that trypanosome antigens (with both *T. b. gambiense* and *T. b. rhodesiense* infections) appeared in the blood soon after infection [10], [11] were present at detectable levels throughout the infection (regardless of the oscillating parasite population associated with antigenic variation) and were reduced to undetectable levels within weeks of the infections being cured [12], [13]. Despite reports of antigen detection assays for animal and human trypanosomiasis [7], [8], [9], [10], [11], [12], [13], [14], [15] only one (for animal trypanosomiasis; [9]) described the identity of the analyte. No reliable antigen detection tests have been developed and implemented for wide-scale use in the field. The collective data suggest that antigen detection assays have potential for diagnosis and monitoring of HAT although it is clear that more effort is required to identify parasite antigens of greatest utility. Several strategies towards antigen identification have been publically suggested [16], [17]. However these are based on examination of the parasites themselves and are not aimed directly at identification of the most relevant molecules that are found circulating in a patient’s bloodstream. Identification of parasite proteins in the blood or plasma of an infected host is the most direct approach for discovery of candidate biomarkers for diagnosis and monitoring of trypanosomiasis. This strategy, however, is made technically difficult by the high abundance of human plasma proteins [18], [19], almost certainly explaining the failure over the past 30 years to identify trypanosome antigens in the blood of infected patients.

Here we describe a “deep mining” protein discovery methodology using liquid chromatography-tandem mass spectrometry (LC-MS/MS) to identify low abundance trypanosome proteins in human plasma. The approach used involved immunodepletion of the most abundant human plasma proteins, protease digestion of remaining proteins, extensive fractionation of the peptides and their identification using a highly sensitive Q Exactive Orbitrap mass spectrometer (Thermo Scientific). The peptides were used to identify their parent proteins, both human and trypanosome. To test this approach, we used plasma from. *T. b. rhodesiense*-infected patients with late stage sleeping sickness, since this parasite can cause acute infections with high levels of parasitemia, thus potentially releasing detectable levels of parasite proteins into the blood. Trypanosome proteins identified by this immunodepletion-proteomic technique are candidate biomarkers and ideal targets for developing new immunoassays assays for either initial diagnosis or for monitoring HAT. Although our initial study was performed with plasma from late-stage patients infected with *T. b. rhodesiense*, the results indicate that the generic approach and methods could be applied to *T. b. gambiense* infections, to animals infected with other trypanosome species and to many other infectious diseases.

## Materials and Methods

### Ethics Statement

Ethical clearance for the project “Biomarker discovery for staging human African trypanosomiasis patients” involving human plasma and CSF from sleeping sickness patients and control subjects was approved by the Ethical Review Board of the Vector Control Division of the Ministry of Health, Kampala, Uganda. All sleeping sickness patients gave written informed consent before enrolment and all had the option of withdrawing from the studies at any time. The Human Research Ethics Board of the University of Victoria (protocol number: 11–531) and the Massachusetts Institute of Technology’s Committee on the Use of Humans as Experimental Subjects, one of the Broad Institute’s institutional review boards of record, also approved the studies.

### Trypanosomes and Cell Culture

*T. congolense* IL3000 [20], *T. b. brucei* 427.01 [21], *T. b. rhodesiense* ViTat 1.1 [22] and *T. b. gambiense* U2 [22] were originally obtained as bloodstream form (BSF) stabilates from the International Livestock Research Institute, Nairobi, Kenya. Procyclic culture forms (PCF) of trypanosomes were derived from their corresponding BSF by transformation at 27°C [23] and were maintained at 27°C in a modified minimal essential medium containing 10% fetal bovine serum [20].

### Production of Anti-trypanosome Antibodies

As probes for parasite antigens, anti-trypanosome antibodies were generated by immunizing rabbits with lysates of *T. b. rhodesiense* PCF. The PCF life cycle stage was chosen as the immunogen in order to generate antibodies against conserved cellular proteins and to avoid reactivity with the immunodominant VSG molecules of BSF trypanosomes. Rabbit antisera were made by immunizing two New Zealand White rabbits (contracted to Epitomics Inc., Burlingame CA) with *T. b. rhodesiense* ViTat1.1 PCF lysate. In brief, rabbits were bled to obtain pre-immunization sera and after one day of rest were immunized with trypanosome lysate prepared by sonicating 2×10^7^ parasites/mL in PBS containing 1x protease inhibitor cocktail V (Cat. No. 539137, Calbiochem). An extended immunization procedure over a total of 78 days, involving 4 injections (1 priming dose of immunogen in Freund’s Complete Adjuvant and 3 boosts with immunogen in Freund’s Incomplete Adjuvant) of immunogen preparation was performed. Blood was collected and sera prepared one week after the final boost and stored at −20°C until used.

### Titration of Anti-trypanosome Antibodies

Indirect enzyme linked immunosorbent assays (ELISA) were performed essentially as previously described [24] with the rabbit anti-*T. b. rhodesiense* PCF antiserum used as the source of primary antibodies. The antigens used to coat the ELISA plate wells were PCF lysates (5×10^5^ cells per well of *T. b. rhodesiense* ViTat 1.1, *T. b. gambiense* U2, *T. congolense* IL3000 and *T. b. brucei* 427.01) and diluted human plasmas. Small amounts of plasma samples from patients were used (1 µL plasma diluted in 99 µL of water) to coat the ELISA plate wells in order to avoid overloading the ELISA wells with protein which could either mask low abundance parasite proteins or lead to erroneously high signals resulting from non-specific protein-protein interactions. The secondary antibody used was a 1∶4000 dilution of goat anti-rabbit IgG (H+L) – AP (Cat. No. 075–1516, Kirkegaard-Perry Laboratories, Gaithersburg MD).

### Collection of Plasma and CSF from Human African Trypanosomiasis Patients

Plasma and CSF were collected from sleeping sickness patients received at the Lwala Hospital, Kaberamaido District, Uganda in 2008. Patients were first identified by passive surveillance by the clinician on duty who referred them for parasitological analysis in the hospital laboratory. First, wet blood smears were examined for trypanosomes by light microscopy. For detection of low level parasitemias, the hematocrit centrifugation technique was used [25]. Patients with detectable bloodstream parasitemias were staged after double centrifugation of CSF and microscopic counting of trypanosomes and white blood cells. Blood samples were collected and plasma prepared using Vacutainer tubes (K_2_EDTA; lavender; Becton Dickinson). Following melarsoprol treatment and apparent cure, a second plasma sample was also collected from each patient. All samples were assigned a patient code (e.g. LWO24). An “A” or “B” following the patient code indicates that the sample was collected either while the patient was infected or after drug cure, respectively.

All plasmas and CSF samples centrifuged and then stored in liquid nitrogen on site at the Lwala Hospital prior to transportation to Makerere University, Kampala, followed by shipment on dry ice to the University of Victoria. A descriptive summary of the HAT samples is shown in Table 1.

**Table 1:** Summary of patient information.

Sample Code	Sex	Age	Date of Isolation	Infection Status	Stage	Leukocyte count	Plasma	CSF
LWO24A	F	55	14/01/2008	*T. b. rhodesiense*	Late	7	Yes	Yes
LWO24B	F	“”	26/03/2008	Drug cured	NA	NA	Yes	No
LWO25A	M	12	17/01/2008	*T. b. rhodesiense*	Late	13	Yes	Yes
LWO25B	M	“”	10/03/2008	Drug cured	NA	NA	Yes	No
LWO31A	M	54	20/02/2008	*T. b. rhodesiense*	Late	86	Yes	Yes
LWO31B	M	“”	13/04/2008	Drug cured	NA	NA	Yes	No

Plasma and CSF were collected from *T. b. rhodesiense*-infected patients confirmed to have late stage human African trypanosomiasis at Lwala Hospital in central Uganda.

### Immunodepletion and Protease Digestion of Plasma from Sleeping Sickness Patients

Plasma samples from three confirmed late-stage HAT patients infected with *T. b. rhodesiense* were pooled to achieve a total volume of 500 µL. The protein concentration in the pooled plasma sample was determined by bicinchoninic acid assay (Cat. No. 23225, Pierce, Rockford, USA) to be 59 mg/ml. The sample was depleted of high and medium abundance human proteins using two immunoaffinity columns in a serial configuration: a Human IgY14 LC20 column (for removal of the top 14 most abundant proteins) and a Human Supermix LC10 column (SEP000-1KT SEPPRO Depletion Column; Sigma-Aldrich St. Lois, MO). The latter resin has been reported to remove at least 155 proteins, 38% of the plasma proteome in protein number and 94% of plasma protein in mass [26]. The depleted plasma was concentrated to 400 µl using a 15 mL, 3 kDa concentrator (Cat. No. UFC900324 Amicon, Billerica, USA) by centrifuging for 60 minutes at 4000×g. The concentrated plasma was then buffer exchanged and denatured with 12 mL of a 6 M urea/50 mM ammonium bicarbonate solution by centrifuging for 2 hours at 4000×g until the final volume of the concentrate reached 400 µl. The protein yield of the depleted, concentrated plasma was determined by a Coomassie Plus (Bradford) Protein Assay (Cat. No. 23236 Thermo Scientific, USA).

Proteins in the denatured sample were first treated with Lys-C (Cat. No. 129-02541, Wako, Richmond, VA) followed by digestion with sequencing grade modified trypsin (Cat. No. V5280, Promega, Madison, WI) according to an in-solution digestion protocol. In brief, the pooled, protein depleted sample was first incubated for 30 minutes at 37°C in 20 mM dithiothreitol followed by addition of iodoacetamide to a final concentration of 50 mM followed by incubation for an additional 30 minutes at room temperature in the dark. The urea was diluted to 2 M with water prior to a 4 hour digestion with Lys-C at 1∶50 (w/w) enzyme to substrate ratio at 37°C. The urea was further diluted with water to 0.6 M and the pH was adjusted to 8.0 with 1 M Tris base prior to trypsin addition (1∶50 enzyme to substrate ratio) and incubation overnight at 37°C. The reaction was stopped by addition of formic acid (FA) to a final concentration of 1% and the solution was desalted with a 1 cc (30 mg) Oasis HLB reverse phase cartridge (Cat. No. WAT054955, Waters, Milford, USA) conditioned with 3×500 µL acetonitrile (ACN), followed by 4×500 µL 0.1% FA. Samples were loaded onto the cartridges and washed with 3×500 µL 0.1% FA. Desalted peptides were eluted by 2 applications of 500 µL of 80% ACN/0.1% FA. Eluates were frozen, dried via vacuum centrifugation and stored at −80°C prior to peptide fractionation.

### Mass Spectrometric Analysis of Peptides from HAT Plasma

Basic (pH 10) reverse phase high-performance liquid chromatography (HPLC) was used to fractionate peptides from all 100 µg of the original digested protein sample using a narrow-bore 2.1×150 mm capillary reverse phase column (Agilent: ZORBAX) packed with 3.5 µm beads and coupled to an Agilent 1100 HPLC system. The mobile phases were as follows: mobile phase A (20 mM ammonium formate, 2% ACN, pH 10) and mobile phase B (90% ACN, 10% 20 mM ammonium formate, pH 10). The gradient was 0–5 min 0% solvent B, 5–55 min 50% solvent B, 55–61 min 100% solvent B, 61–80 min 0% solvent B at a constant flow rate of 0.2 mL/min. A total of 30 fractions were collected over 0–80 minutes. The fractions were frozen at −80°C and dried before LC-MS/MS analysis.

The peptides samples in each of the 30 fractions were resuspended in 8 µL of 3% ACN/0.5% FA before analysis using a Q Exactive mass spectrometer coupled to an EASY-nLC 1000 UHPLC (Thermo Scientific). A PicoFrit column (New Objective, Woburn, MA), with an inner diameter of 75 µm and packed with 20 cm of ReproSil-Pur C18 1.9 µm particles, was directly interfaced to the Q Exactive instrument equipped with a custom nano-electrospray ionization source. Two µg of the peptide mixture from each of the 30 fractions were injected and separated by a 180 minute gradient from 5–60% solvent B. MS/MS analysis settings for protein identification were as follows: one precursor MS scan at 70,000 resolution in profile mode was followed by data-dependent scans of the top 12 most abundant ions at low-resolution (17,500) in profile mode. Dynamic exclusion was enabled for a duration of 20 seconds. MS/MS spectra were collected with a normalized collision energy of 28 and an isolation width of 2.5 m/z. The extensive peptide fractionation coupled with in depth MS analysis (instrument time was 90 hours) allowed detection and identification of very low levels of peptides.

### Data Analysis

All MS data were processed using Agilent Spectrum Mill MS Proteomics Workbench (Agilent Technologies, Palo Alto, USA) Rev B.04.00.120. MS/MS spectra were searched against an amalgamated human protein database (UNIPROT) and a *T. b. brucei* protein database: (http://phenyx.proteomics.washington.edu/FASTAcreator/index.cgi). Search parameters were set to: parent mass tolerance of 20 ppm, fragment mass tolerance of 30 ppm, a maximum of two missed cleavages and carbamidomethylation and oxidized methionine/pyroglutamic acid as fixed and variable modifications, respectively. Database matches for individual spectra were auto-validated according to user-defined scoring thresholds for both peptides (false discovery rate (FDR) ≤1.2%) and proteins (minimum protein score of 20) in a two-step process. In calculating scores at the protein level and reporting the identified proteins, redundancy was addressed in the following manner: the protein score is the sum of the scores of distinct peptides. A distinct peptide is the single highest scoring instance of a peptide detected using a MS/MS spectrum. MS/MS spectra for a particular peptide may have been recorded multiple times (i.e. as different precursor charge states, isolated from adjacent reverse phase fractions, oxidation of Methionine) but are still counted as a single distinct peptide. In Spectrum Mill, FDRs are calculated at 3 different levels: spectrum, distinct peptide and distinct protein. Peptide FDRs are calculated in Spectrum Mill using essentially the same pseudo-reversal strategy evaluated by Elias et al. [27] and shown to perform the same as concatenation. A false distinct protein identification occurs when all of the relevant peptides, which group together to constitute a distinct protein, have a delta Forward Reverse Score ≤0. Spectrum Mill also performs protein grouping using the methods described by Nesvizhskii and Aebersold [28]. Briefly, when a peptide sequence, (>8 residues long) is contained in multiple protein entries in the protein database, the proteins are grouped together and the highest scoring one and its accession number are reported. In some cases when the protein sequences are grouped in this manner there are distinct peptides which uniquely represent a lower scoring member of the group (isoforms and family members). Each of these instances spawns a subgroup and multiple subgroups are reported and counted towards the total number of proteins.

The sum of the precursor-ion signal intensities of all peptides derived from each protein was used as an approximation of that protein’s expression level. The peak area for the extracted ion chromatogram of each precursor ion in the intervening high-resolution MS scans of the data-dependent LC-MS/MS runs was calculated automatically by the Spectrum Mill software using narrow windows around each individual member of the isotope cluster. Peak widths in both the time and m/z domains were dynamically determined based on MS scan resolution, precursor charge and m/z subject to quality metrics on the relative distribution of the peaks in the isotope cluster vs theoretical. For a given protein the ratio of the total protein intensity to total unique peptides can be deemed the “molar intensity” of that protein. Therefore, a ranking of the protein molar intensity from large to small values should, within an order of magnitude, rank the abundance of the proteins within the given sample.

## Results and Discussion

### Characterization of Plasma from HAT Patients

**Figure 1:**
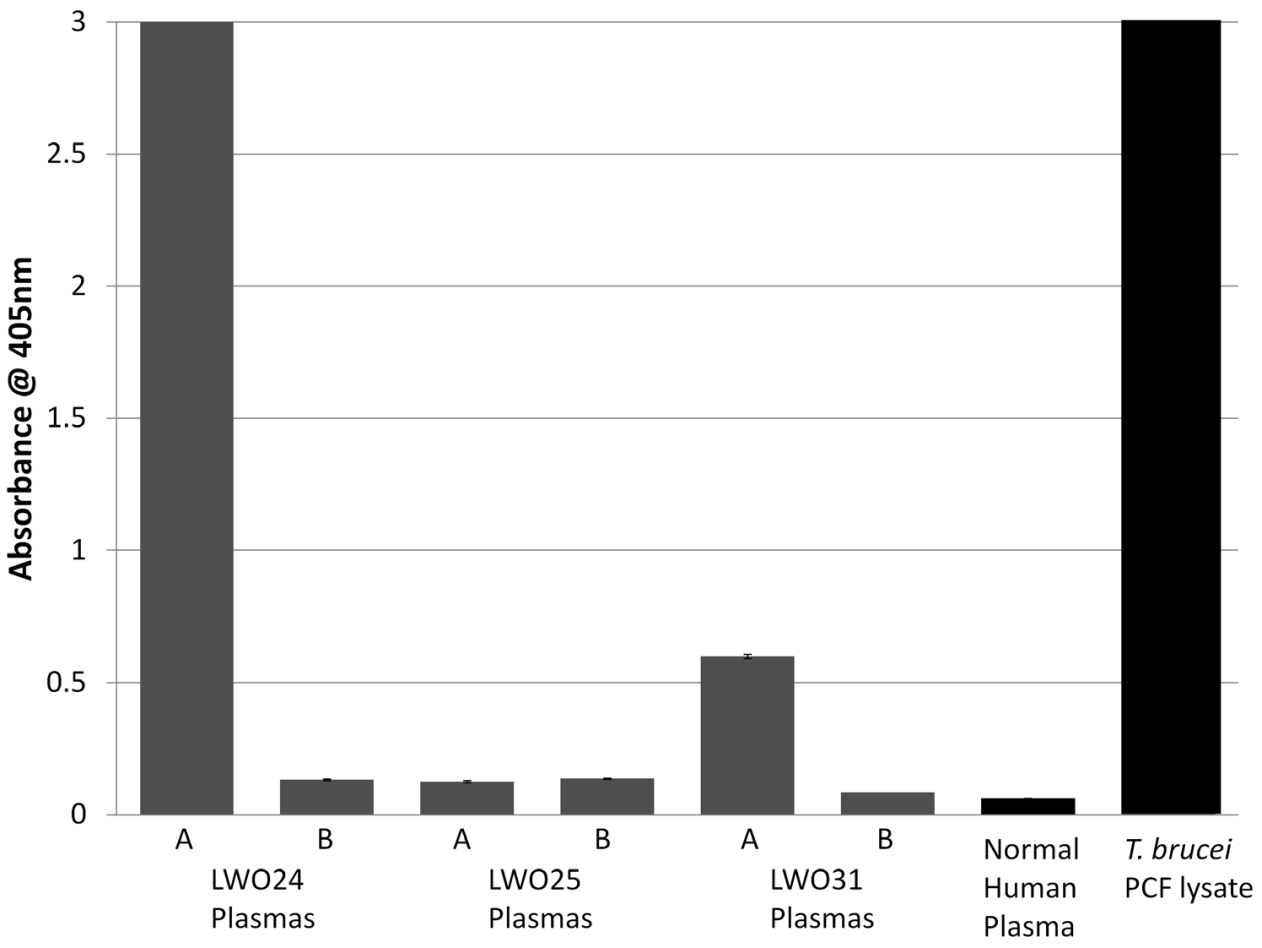
ELISA detection of trypanosome antigens in plasma from *T. b. rhodesiense* -infected patients with late-stage human African trypanosomisis. All samples were tested in duplicate and the average response is shown. Error bars represent 1 standard deviation.

To recapitulate previous literature reports, and to gain confidence that trypanosome antigens were present prior to undertaking our extensive MS analysis, plasma samples from HAT patients were examined for the presence of conserved, trypanosome antigens by indirect ELISA using polyclonal antiserum against *T. b. rhodesiense* PCF as a probe. The anti-trypanosome antibodies were first characterized by immunoblotting and ELISA and reacted in both assays with antigens in lysates of the immunizing *T. b. rhodesiense* PCF and in lysates of *T. congolense*, *T. b. brucei*, and *T. b. gambiense* (data not shown). The results of the ELISA performed on human plasma are shown in Figure 1. Two patients (LWO24 and LWO31) had readily detectable trypanosome antigens in their plasmas that were recognized by the anti-PCF antiserum whereas one patient (LW025) did not. The antigens were not detected in plasmas taken after drug cure and in plasma from a normal (uninfected) control. Trypanosome antigens were also detected in CSF of patient LW024 (data not shown). Since the antigens detected by the anti-*T. b. rhodesiense* PCF antiserum may only represent a subset of the antigens present in the trypanosome infected plasmas, we chose to pool the samples from all three patients prior to the immunodepletion-MS approach to be used in the current study. These three patients provided a good cross section of the patient population. One patient (LWO24) was a middle aged female. Of the two males, one was middle aged (LWO25) and the other was in his teens (LWO31). Despite the plasmas showing variations in antigen levels by immunoassay with the polyclonal anti-PCF antibodies used, all three patients were parasitologically confirmed to have late stage African sleeping sickness. The polyclonal antiserum was only used in ELISA to generally assess the presence of trypanosome antigens (described in several previous publications) in the plasma samples and there is no guarantee that the antigens identified by our MS analysis are the same as those detected by immunoassay.

### Identification of Trypanosome Proteins in Human Plasma

The pooled plasma sample was first immunodepleted of high- and medium-abundance human proteins to allow detection of the remaining low-abundance proteins, hopefully including those from trypanosomes. One hundred µg of total protein (from an initial 29.5 mg in the 500 µL of pooled plasma) remained after protein depletion and concentration, representing 0.34% of the initial protein content. After protein digestion to tryptic peptides, fractionation into 30 basic reverse phase fractions and analysis of each fraction by LC-MS/MS, 254 trypanosome proteins were confidently identified (*i.e.* with at least 2 contributing peptides). The results are shown in the Supporting Information File (Appendix S1). An additional 570 trypanosome proteins were identified based on detection and sequencing of single unique peptides, bringing the total number of identified trypanosome proteins to 824. The protein identifications made on the basis of single peptides may still be relevant but the identifications are considered to be less reliable than those identifications made on the basis of multiple observed peptides. Nevertheless, the single peptides represent non-human sequences and map to trypanosome proteins thus are deemed to be of parasite origin. It is possible that some trypanosome proteins could have been removed inadvertently by off-target binding during the immunodepletion step [26] although clearly many parasite proteins remained at high enough levels for their positive identification.

In addition to the trypanosome proteins, 4326 normal human proteins were also confidently identified (based on two peptides) and a further 1843 human proteins were possibly correctly identified (based on one peptide), demonstrating that the combined depletion-MS method was extremely effective in identifying a large variety of low abundance proteins in plasma. The sensitivity achieved with the combined methods for detection of trypanosome proteins suggests that parasite proteins may be detected even with much lower levels of parasitemia, for example at early stages of infection with *T. b. rhodesiense* or with the usually more chronic infections with *T. b. gambiense*.

A complete list of all of the proteins identified (both human and trypanosome) can be found in the Supporting Information as an Excel file (Appendix S1). The single peptide data have also been included in Appendix S1 but will not be discussed further in the text of this manuscript.

The large number of trypanosome proteins discovered in the plasma from the HAT patients can be attributed in part to our choice of plasmas from patients with late-stage *T. b. rhodesiense* infections, part of our strategy to ensure that parasite proteins were present for this proof of concept approach. Although specific durations of infection prior to hospitalization for these patients were unknown it has been established that near Lwala, Uganda, the late stage of HAT is reached, on average, 4 weeks after infection with *T. b. rhodesiense* [29]. Thus presumably, all patients had experienced several waves of parasitemia, potentially allowing trypanosome proteins to accumulate in the bloodstream after secretion or shedding from live parasites or released upon parasite destruction mediated by the host immune system. In addition, the identification of trypanosome proteins was facilitated by depletion of the highly- and moderately-abundant human proteins, the long reverse phase HPLC peptide fractionation (30 fractions collected over 80 min) and extensive MS analysis (3 hours of MS time per fraction for a total instrument time of 90 hours) using a highly sensitive Q Exactive Orbitrap mass spectrometer. Too much data were generated in our MS experiments to be described in detail here, therefore only some of the interesting groups of trypanosome proteins will be discussed.

To our knowledge, there has been only one previously published report describing unbiased discovery of proteins from any pathogen in human body fluids (a single protein identified with 2 contributing peptides in the saliva of malaria patients [30]), although targeted assays have been created and used to detect preselected parasite proteins (from trypanosomes and plasmodia) in mammalian fluids [9], [31]. By using an unbiased discovery method, it is more likely that those trypanosome proteins which are abundant, conserved, soluble and persistent in human plasma will be identified. Our results support this idea.

Surprisingly, trypanosome VSGs immediately stood out as one of the most conspicuous groups of proteins identified in the pooled plasma. It was interesting to find twelve highly divergent trypanosome VSG sequences (Table 2). The antibody response of infected hosts is known to be directed against the VSG coat presented on the dominant antigenic types of trypanosomes of each parasitemic wave and it has long been believed that within each wave (at least at early time points in an infection) a single VSG type dominates. It has often been assumed that the predominant, abundant and highly immunogenic VSGs would be cleared from the body rapidly after parasite phagocytosis or complement mediated lysis. In the plasma sample used here (a pool from three patients), it would be expected that fewer VSG types would be seen, perhaps those from the most recent parasitemic wave. The identification of such a wide variety of VSGs was unexpected and of interest for understanding the infection process and our current model of antigenic variation. We speculate that either the VSGs persist in the bloodstream with successive parasitemic waves, or the waves of parasitemia become unsynchronized with parasites expressing multiple different VSG types late in infection. All the patients used here had late stage infections so either explanation is possible. Even if our conception of antigenic variation is compatible with multiple persistent VSGs, the results indicate that this deep-mining proteomics method is a powerful tool to obtain a snapshot of the VSG types present in infected patients and to study the temporal unfolding of the disease process. We are not aware of any other method that could achieve this type of analysis. The only other membrane protein confidently observed in the data set was the kinetoplastid membrane protein 11 (KMP-11, Accession number Tb09.211.4511) that has been identified in the secretome of *T. congolense* BSF [32].

**Table 2:** Variant Surface Glycoproteins (VSGs) discovered in pooled plasma from *T. b. rhodesiense*-infected patients confirmed to have late-stage human African trypanosomiasis.

Molar Intensity	Accession No.	No. of Peptides	% Sequence Coverage	Protein Name
3.49E+09	Tb927.5.291b	2	5.1	variant surface glycoprotein, putative
9.80E+08	Tb09.244.0200	3	5.4	variant surface glycoprotein, putative
9.10E+08	Tb10.v4.0152	2	3.5	variant surface glycoprotein, putative
5.20E+08	Tb11.57.0012	2	5.3	variant surface glycoprotein pseudogene, putative
4.97E+08	Tb927.5.5210	2	6.6	variant surface glycoprotein, putative
4.46E+08	Tb11.30.0008	2	4	variant surface glycoprotein pseudogene, putative
2.58E+08	Tb927.5.4670	5	14.4	variant surface glycoprotein, putative
2.27E+08	Tb927.4.5410	2	5.3	variant surface glycoprotein, putative
2.11E+08	Tb10.v4.0058	2	5.2	variant surface glycoprotein, putative
1.71E+08	Tb05.5K5.530	2	5.6	variant surface glycoprotein, putative
1.37E+08	Tb05.5K5.320	2	3.8	variant surface glycoprotein, putative
6.85E+07	Tb10.v4.0134	2	4.8	variant surface glycoprotein, putative

The largest groups of annotated trypanosome proteins identified were those involving protein folding (chaperones and isomerases, Table 3) and protein degradation (proteases, peptidases and ubiquitin related proteins, Table 4). This can possibly be explained by the high rate of cell division and VSG synthesis or recycling in BSF trypanosomes. Although these proteins have less of a direct apparent impact on the disease process (compared to the well known VSGs), the identification of so many of this group (23 chaperones and 18 proteases/ubiquitin related proteins) raises questions as to why they persist in the bloodstream and whether or not they play a role in the disease process. Beyond the known functions of the folding proteins and proteases inside the trypanosome, it is possible that some (especially the proteases) have a function once released by the parasites. It is easy to envision trypanosome derived proteases acting as virulence factors when released into the bloodstream. Indeed a well-studied trypanosome protease, congopain, has been hypothesized to act as a virulence factor [33]. The data that we have generated here may offer supporting evidence to justify future research into the topic.

**Table 3:** Chaperones and protein isomerases identified in pooled plasma from *T. b. rhodesiense*-infected patients with parasitologically confirmed late stage human African trypanosomiasis.

Molar Intensity	Accession No.	No. of Peptides	% Sequence Coverage	Protein Name
3.08E+10	Tb927.7.710	4	6.7	heat shock 70 kDa protein, putative
2.65E+10	Tb927.6.3740	4	8.6	heat shock protein 70, mitochondrial precursor, putative
1.73E+10	Tb10.61.1940	2	2.2	chaperone protein DNAJ, putative
1.52E+10	Tb09.211.1350	2	6.7	peptidyl-prolyl cis-trans isomerase, putative
8.33E+09	Tb11.01.3110	33	47.9	heat shock protein 70
6.10E+09	Tb927.7.4590	2	2.1	chaperone protein DNAJ, putative
4.96E+09	Tb927.7.4770	8	64.7	peptidyl-prolyl cis-trans isomerase, putative
4.79E+09	Tb10.26.1080	29	40.3	heat shock protein 83
4.22E+09	Tb927.7.1320	6	77	1010 kDa heat shock protein, putative
3.89E+09	Tb11.03.0250	10	74	cyclophilin a
2.05E+09	Tb927.7.5790	4	25.1	protein disulfide isomerase, putative
1.81E+09	Tb11.02.5450	11	23.5	glucose-regulated protein 78, putative
1.69E+09	Tb10.6k15.2290	10	22.5	protein disulfide isomerise
1.49E+09	Tb927.8.7410	17	50.6	calreticulin, putative
1.49E+09	Tb927.4.5010	16	45	calreticulin, putative
1.12E+09	Tb10.61.0180	9	38.5	peptidylprolyl isomerase-like protein, putative
2.86E+08	Tb927.7.1300	13	39.5	protein disulfide isomerase, putative
2.47E+08	Tb10.70.0280	10	26.3	heat shock protein 60 chaperonin, mitochondrial precursor
2.07E+08	Tb927.8.690	2	26.9	peptidyl-prolyl cis-trans isomerase, putative
1.39E+08	Tb927.5.2940	10	20.9	stress-induced protein sti1, putative
9.19E+07	Tb10.389.0880	9	17	heat shock protein, putative
8.05E+07	Tb927.3.5340	2	6.2	heat shock cognate 70 - interacting protein, putative
6.35E+07	Tb11.02.0250	8	12.2	heat shock protein 84, putative

**Table 4:** Proteases and ubiquitin proteins discovered in pooled plasma from *T. b. rhodesiense*-infected patients with parasitologically confirmed late stage human African trypanosomiasis.

Molar Intensity	Accession No.	No. of Peptides	% Sequence Coverage	Protein Name
5.43E+10	Tb11.01.1680	12	9.4	polyubiquitin, putative
2.90E+09	Tb09.211.3610	5	3.9	ubiquitin-activating enzyme E1, putative
2.70E+09	Tb09.211.0050	5	23.3	ubiquitin-conjugating enzyme E2, putative
2.08E+09	Tb927.5.1000	5	68.9	ubiquitin-conjugating enzyme E2, putative
1.68E+09	Tb927.2.3030	2	1.8	ATP-dependent Clp protease subunit, putative
1.48E+09	Tb11.47.0035	2	0.4	calpain-like cysteine peptidase, putative
1.44E+09	Tb11.02.0070	3	9.7	aminopeptidase NPEPL1
9.67E+08	Tb11.02.4440	24	70.8	aminopeptidase, putative
7.17E+08	Tb927.7.4060	6	60	calpain-like cysteine peptidase, putative
6.30E+08	Tb11.02.0815	2	18.1	ubiquitin-conjugating enzyme, putative
3.38E+08	Tb10.6k15.2520	34	56.3	prolyl oligopeptidase, putative
1.25E+08	Tb10.6k15.0580	2	4.6	RPT6 proteasome regulatory ATPase subunit 6
9.93E+07	Tb10.6k15.3800	3	3.5	dipeptidyl-peptidase 8-like serine peptidase
9.80E+07	Tb10.70.7080	2	10.3	serine carboxypeptidase III precursor, putative
8.25E+07	Tb927.6.2150	2	4	cell division cycle protein 16, putative
7.73E+07	Tb927.7.190	4	6.4	oligopeptidase A, putative
5.77E+07	Tb11.02.0100	3	8.1	carboxypeptidase, putative
4.66E+07	Tb10.61.1870	2	6.2	aminopeptidase, putative

In addition to the protein families mentioned above, many metabolic, cytoskeletal, and signaling proteins of trypanosomes were identified in human plasma. A further 92 trypanosome proteins were annotated as uncharacterized “hypothetical proteins”, highlighting the understudied nature of these parasite proteins.

Many of the trypanosome proteins in plasma from HAT patients are potentially interesting for understanding the biology and biochemistry of disease. However, the abundant, conserved proteins are perhaps the most important from an applied point of view. Identification of trypanosome proteins in plasma may allow selection of those that might be most useful as biomarkers for diagnosis and monitoring of African sleeping sickness or animal trypanosomiasis. A first step might be to determine which are the most abundant parasite proteins in plasma. However, unlike several other mass spectrometry techniques such as stable isotope standards and capture by anti-peptide antibodies (SISCAPA) [34] or isobaric tags for relative and absolute quantitation (iTRAQ), that has already been used to study protein expression in trypanosomes [35], the protein discovery method described here has no built-in quantitation capability. Therefore, in order to gain a rough measure of protein abundance, the trypanosome proteins were assigned a “molar intensity” score which was calculated by averaging the total ion intensity for a given protein by the number of peptides identified from that protein. This value represents total ion intensity per peptide. If it is assumed that a mole of peptide (regardless of source or chemical nature) gives rise to the same level of signal intensity, then molar intensity can be used as a means to rank protein abundance. However, this method is flawed, because it is known that the mass, charge and physicochemical properties influence peptide behaviour and thus signal intensity in a mass spectrometer. Nevertheless, we judge that this method can be used to rank proteins with an accuracy of approximately one order of magnitude. We have used this approach to select the 15 most abundant trypanosome proteins (Table 5). The proteins in this table, not surprisingly, are conserved soluble proteins and represent a broad spectrum of functional roles. Six of the fifteen most abundant trypanosome proteins are chaperones or proteases/ubiquitin related proteins. The rest are a collection of enzymes, motor proteins, signaling proteins and two proteins of unknown function (a hypothetical protein and a retrotransposon hot spot protein). Some of these may be of sufficient abundance to be of used as biomarkers for diagnosis and monitoring of trypanosome infections using sandwich immunoassays or other protein detection techniques. While it is unknown by which route these proteins entered the human bloodstream (shedding/secretion or lysis/necrosis), it is interesting to note that six of the abundant proteins listed in Table 5 (chaperone protein DNAJ, peptidyl prolyl cis-trans isomerase, triose phosphate isomerase and three forms of heat shock protein 70) were also identified in the secretome of cultured *T. congolense* BSF [32].

**Table 5:** Most abundant* trypanosome proteins found in pooled plasma from *T. b. rhodesiense*-infected patients with parasitologically confirmed late-stage human African trypanosomiasis.

Molar Intensity	Accession No.	No. of Peptides	% Sequence Coverage	Protein Name
5.43E+10	Tb11.01.1680	12	9.4	polyubiquitin, putative
3.08E+10	Tb927.7.710	4	6.7	heat shock 70 kDa protein, putative
2.65E+10	Tb927.6.3740	4	8.6	heat shock 70 kDa protein, mitochondrial precursor, putative
2.13E+10	Tb927.2.1170	2	2.2	retrotransposon hot spot protein, putative
1.73E+10	Tb10.61.1940	2	2.2	chaperone protein DNAJ, putative
1.63E+10	Tb927.8.5600	3	16.2	transaldolase, putative
1.52E+10	Tb09.211.1350	2	6.7	peptidyl-prolyl cis-trans isomerase, putative
1.42E+10	Tb11.02.3210	11	57.2	triosephosphate isomerase
1.37E+10	Tb11.01.4621	13	61.7	calmodulin
1.11E+10	Tb10.6k15.3410	2	1.6	pre-mRNA splicing factor ATP-dependent RNA helicase, putative
9.30E+09	Tb927.4.5340	2	2.2	hypothetical protein, conserved
9.27E+09	Tb927.8.3250	3	0.5	dynein heavy chain, putative
9.20E+09	Tb10.70.4880	2	3.4	eukaryotic translation initiation factor 5, putative
8.73E+09	Tb927.5.2090	3	1.7	kinesin, putative
8.33E+09	Tb11.01.3110	33	47.9	heat shock protein 70

* Abundant proteins were determined by calculating molar intensities as described in Materials and Methods.

To obtain an estimate of their concentrations in plasma, the molar intensities of the 15 most abundant trypanosome proteins were compared with the intensities of normal human proteins, also identified in our experiment, for which literature reported concentration values are available [19]. Table 6 lists the normal human proteins (identified in the HAT plasma) that had molar intensities similar to those from the most abundant trypanosome proteins. By comparison to the normal human proteins, it appears that the most abundant trypanosome proteins exist in the bloodstream in the range of 5–40 µg/mL although again these are crude approximations from a pooled sample. Nevertheless, this puts these proteins well within detection range of standard diagnostic immunoassays and SISCAPA assays [34].

**Table 6:** List of human plasma proteins with mass spectrometric molar intensities similar to those from the most abundant trypanosome proteins found in plasma from sleeping sickness patients.

Molar Intensity	Accession No.	No. of Peptides	% Sequence Coverage	Concentration (µg/ml)*	Protein Name
5.01E+10	P22352	15	58.8	19.0	glutathione peroxidase 3
4.17E+10	P18428	19	38	5.0	lipopolysaccharide-binding protein
3.93E+10	P25311	28	71.4	45.0	zinc-alpha-2-glycoprotein
3.56E+10	P36955	34	64.8	5.0	pigment epithelium-derived factor
2.48E+10	P07225	33	51.7	25.0	vitamin K-dependent protein S
1.69E+10	P29622	31	67.6	14.0	kallistatin
1.53E+10	P04278	25	80	6.5	sex hormone-binding globulin
1.38E+10	P03951	40	73.4	5.0	coagulation factor XI
1.30E+10	Q96IY4	22	57.4	9.5	carboxypeptidase B2
1.18E+10	P23142	29	51.3	40.0	fibulin-1
9.18E+09	P17936	17	58.4	5.0	insulin-like growth factor-binding protein 3
9.01E+09	P04275	142	61	15.0	von Willebrand factor

* Protein concentrations were retrieved from reference [19]. Human proteins reported to be depleted by the LC20 IgY14 and Supermix LC10 columns [26] have been omitted from this table.

The sample processing and analysis approach developed at the Broad Institute enabled the confident identification of 4326 distinct human proteins with at least 2 peptides/protein and an additional 1843 proteins with lower confidence (for a total of 6169 proteins) if single peptide identifications are included. This equates to approximately 21% of the predicted human proteome [36], or ca. 30% if single peptide hits are also considered. This represents one of the largest sets of human plasma proteins reported from a single MS experiment in the published literature and attests to the efficacy of the immuno-depletion method coupled with extensive peptide fractionation and sensitive MS detection.

While some of the trypanosome proteins discovered in plasma may be useful as biomarkers, their identification here is based on a single, painstaking, extensive and expensive experiment. Nevertheless, the unequivocal peptide identification data provide strong proof of principle for the discovery of parasite proteins in human body fluids. The next step will be to perform a similar analysis using plasma from patients with early stage HAT, including those chronically infected with *T. b. gambiense* parasites, to identify proteins that appear early in the infection process and that are conserved between *T. brucei* subspecies. In this way, protein antigens may be identified for development of sandwich immunoassays or SISCAPA assays [34] and validation of the biomarkers for diagnostic utility using plasma or sera from many patients.

To our knowledge, this study reports the first identification of microbial proteins in the human bloodstream using a unbiased approach, that is, without the use of an assay designed to specifically detect predetermined analytes. Previous application of mass spectrometric approaches to diagnosis of trypanosomiasis used methods that do not allow identification of the relevant molecules [37], [38].

African trypanosomiasis caused by *T. b. rhodesiense* may be ideally suited for the protein discovery methodology described here because of the high parasite burden experienced by patients infected with this parasite. In addition, plasma from patients with late stage infections perhaps contained high levels of trypanosome proteins since antigenic variation results in sequential and extended lysis of parasites, leading to a relatively high concentration of released parasite material. However, our results showing detection of low abundance proteins imply that the methods should be effective for biomarker discovery even with infections showing much lower parasite burdens, for example with *T. b. gambiense*. Although infections with African trypanosomes may be suited to the biomarker discovery method outlined here, the great sensitivity achieved with the general approach bodes well for studying the infection proteomics of a variety of infectious diseases.

## Supporting Information

Appendix S1 **Excel sheet 1 contains a list of all proteins (trypanosome and human) identified in pooled plasma from human African trypanosomiasis patients and listed by number of contributing peptides.** Excel sheet 2 shows only the trypanosome proteins discovered in plasma from patients with human African trypanosomiasis. Excel sheet 3 shows only the trypanosome proteins (with at least 2 contributing peptides) organized by putative function.
(XLSX)
